# Covalent bonding of heme to protein prevents heme capture by nontypeable *Haemophilus influenzae*

**DOI:** 10.1002/2211-5463.12324

**Published:** 2017-10-12

**Authors:** Valerie Sgheiza, Bethany Novick, Sarah Stanton, Jeanetta Pierce, Breanne Kalmeta, Melody Frink Holmquist, Kyle Grimaldi, Kara L. Bren, Lea Vacca Michel

**Affiliations:** ^1^ School of Chemistry and Materials Science Rochester Institute of Technology NY USA; ^2^ Department of Chemistry University of Rochester NY USA

**Keywords:** cytochrome *c*, *Haemophilus influenzae*, heme

## Abstract

Nontypeable *Haemophilus influenzae* (NTHi) are Gram‐negative pathogens that contribute to a variety of diseases, including acute otitis media and chronic obstructive pulmonary disease. As NTHi have an absolute requirement for heme during aerobic growth, these bacteria have to scavenge heme from their human hosts. These heme sources can range from free heme to heme bound to proteins, such as hemoglobin. To test the impact of heme structural factors on heme acquisition by NTHi, we prepared a series of heme sources that systematically vary in heme exposure and covalent binding of heme to peptide/protein and tested the ability of NTHi to use these sources to support growth. Results from this study suggest that NTHi can utilize protein‐associated heme only if it is noncovalently attached to the protein.

**Abbreviations**
AOM: acute otitis media
BHI: brain heart infusion
COPD: chronic obstructive pulmonary disease
CXXCH: Cys‐X‐X‐Cys‐His
cyt: cytochrome
*E. coli*: *Escherichia coli*
Hib: *Haemophilus influenzae* type b
*Ht*‐C12A: *Ht* C12A cyt *c*
*Ht*‐C15A: *Ht* C15A cyt *c*
*Ht‐*DM: *Ht* C12A/C15A cyt *c*
*Ht*: *Hydrogenobacter thermophilus*
LB: Luria broth
MP‐11: microperoxidase 11
NTHi: Nontypeable *Haemophilus influenzae*
ppIX: protoporphyrin IX

Nontypeable *Haemophilus influenzae* (NTHi) are human commensal organisms that typically live in the nasopharynx, but can cause diseases such as acute otitis media (AOM), sinusitis, conjunctivitis, pneumonia, meningitis, and chronic obstructive pulmonary disease (COPD) 1, 2. For commensals and pathogens living in or invading human tissues, iron is often a limiting nutrient; one iron source for those bacteria is heme, protoporphyrin IX (ppIX) that contains an iron ion at its center 3. As its name suggests (hemophilus means ‘blood loving’ in Greek), NTHi have an absolute requirement for heme during aerobic growth, fulfilled either through direct uptake of heme or through uptake of ppIX followed by iron chelation 4, 5.

To obtain exogenous heme, NTHi have evolved a hemophore‐mediated heme uptake system, which secretes heme‐binding proteins (hemophores) to scavenge heme from host proteins, such as hemoglobin and hemopexin 6, 7. Under heme‐limiting conditions, a complex and redundant system of secreted hemophores and membrane proteins works in concert to deliver heme from the external environment to the cytoplasm of the bacterial cell 4. Secreted hemophores extract heme from the hemoprotein and pass it along to a heme surface receptor or bind directly to the hemoprotein, causing the hemoprotein to release its heme to a surface receptor in a single step (without directly interacting with the heme group) 8. An additional set of transporters is required to move the heme across the cytoplasmic membrane into the cytoplasm 9.

A more detailed mechanism of heme extraction from the heme‐binding protein, hemopexin, was elucidated for NTHi's HxuABC system 10. Hemopexin is present in all mammalian body fluids and exhibits one of the highest known binding affinities to heme 11. During heme acquisition, a conformational change in HxuA induces structural changes in hemopexin, lowering its affinity for heme and resulting in heme release to the HxuC receptor 10. In addition to the HxuABC system, the HemR and Hgp/Hup systems have been linked to free heme acquisition and heme acquisition from hemoglobin in *H. influenzae* type b (Hib), an encapsulated strain of *H. influenzae* 12, 13, 14.

While most studies have focused on hemoproteins such as hemoglobin and hemopexin, one study demonstrated that horse cytochrome (cyt) *c* failed to provide an adequate heme source to Hib 15. Unlike hemoglobin and hemopexin, the heme group in cyt *c* proteins, including human cyt *c* (which shares 89% sequence identity with horse cyt *c*), is covalently attached to the protein via a Cys‐X‐X‐Cys‐His (CXXCH) motif (Fig. 1). Whether horse cyt *c* failed to interact with Hib's hemophore(s) or the hemophore(s) were unable to extract heme due to the covalent linkages was not addressed in that study.

**Figure 1:**
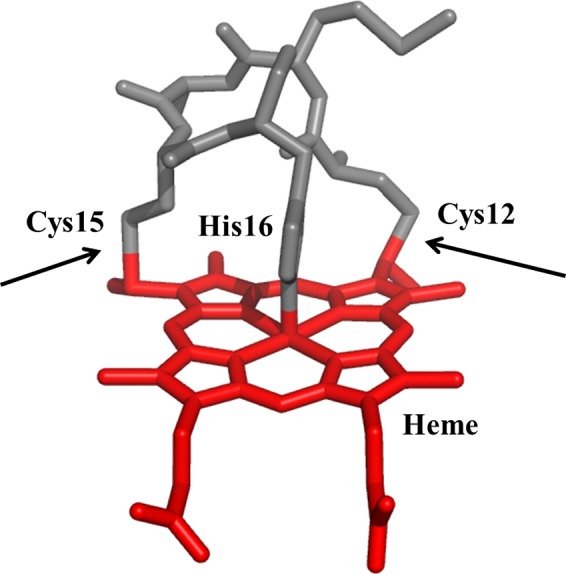
The heme and CXXCH motif from *Hydrogenobacter thermophilus* cyt *c* (Protein Data Bank ID: 1YNR). One or both covalent linkages between peptide and heme (arrows) are absent in the single mutants (*Ht* C12A cyt *c*;* Ht* C15A cyt *c*) and the double mutant (*Ht* C12A/C15A cyt *c*), respectively. The His residue (His16) coordinates to the heme iron.

In this work, we sought to further our understanding of the effect of heme‐binding mode and solvent exposure on heme scavenging by the pathogenic bacteria NTHi. As a heme source, we used single (C12A or C15A) and double (C12A/C15A) site‐directed mutants of *Hydrogenobacter thermophilus* (*Ht*) cyt *c* in which the Cys residues of the C_12_XXC_15_H motif (Fig. 1) are mutated to Ala 16. *Ht* cyt *c* was selected as an ideal model system for this study, because its inherent stability allows the double mutant protein to maintain a folded structure with heme bound in the absence of thioether linkages to heme 17. This property allowed us to determine the effect of covalent attachment on heme availability within one protein scaffold. To determine whether heme that is solvent‐exposed but has covalent bonds to two Cys can serve as a heme source for NTHi, we supplemented NTHi cultures with microperoxidase‐11 (MP‐11), an 11‐mer peptide with covalently bound heme 18. This series of heme sources (*Ht* cyt *c*, its mutants, MP‐11, and hemin) systematically varies in heme exposure and covalent binding to a peptide, providing a means to test the impact of heme structural factors on heme acquisition.

## Materials and methods

### Mutagenesis, expression, and purification

Plasmid pSCH552 (ampicillin resistant) was used as a template for site‐directed mutagenesis of *Ht* cyt *c* 19. The pSCH552 plasmid contains the *Thiobacillus versutus* cyt *c*_550_ signal sequence inserted in frame and upstream of the *Ht* cyt *c* gene, directing excretion to the periplasm for maturation (covalent heme attachment) in *Escherichia coli* (*E. coli*). The two single mutants, *Ht* C12A cyt *c* (*Ht*‐C12A) and *Ht* C15A cyt *c* (*Ht*‐C15A), were prepared using the QuikChange II kit (Agilent, Santa Clara, CA, USA) according to the manufacturer's instructions and confirmed by DNA sequencing. The plasmid pEST202 encoding *Ht* C12A/C15A cyt *c* (*Ht*‐DM) was a gift from Stuart Ferguson 17. *Ht* wild‐type, C12A, and C15A cyts *c* were expressed and purified as previously described for the wild‐type protein 19. *Escherichia coli* BL‐21(DE3) cells were cotransformed with the plasmid harboring the cyt *c* gene and the pEC86 plasmid (chloramphenicol resistant), which contains the cyt *c* maturation genes *ccmABCDEFGH* 20. The pEC86 plasmid was a gift to Kara Bren from Linda Thöny‐Meyer. *Ht*‐DM was expressed and purified as described 17, with the following modifications. The *E. coli* cells were grown on Luria broth (LB) agar plates, and single colonies were used to inoculate 25 mL LB (supplemented with 50 μg·mL^−1^ ampicillin). Small cultures were grown for 16 h (180 rpm, 37 °C) and used to inoculate large cultures (in 1 L LB, 140 rpm, 37 °C). Protein expression was induced with 1 mm IPTG when the optical density of the cultures at 600 nm (OD_600_) reached ~0.6; cultures were incubated for an additional 3 h before harvesting by centrifugation at 5000 ***g*** for 15 min.

### NTHi growth experiments

Nontypeable *H. influenzae* cultures were grown on brain heart infusion (BHI) medium (BD) supplemented with 30 μm NAD^+^ (Sigma, St. Louis, MO, USA) and either water (negative control) or a single heme source. NTHi lack the enzymes required to synthesize the essential cofactor NAD^+^ and therefore must uptake exogenous NAD^+^. NTHi 86‐028NP is a pediatric isolate (a gift from Lauren Bakaletz, The Research Institute at Nationwide Children's Hospital) 21. In the first set of NTHi growth experiments, heme sources were 15 μm hemin (Sigma), 15 μm purified human hemoglobin (Sigma), or 15 μm wild‐type *Ht* cyt *c*,* Ht*‐C12A, or *Ht*‐DM. A small 500 μL aliquot was removed from each 10 mL sample immediately after the supplements were added, but prior to inoculation, to be used as blanks for the OD readings. The cultures were incubated at 37 °C, shaken at 180–200 rpm, and monitored for changes to the OD_490_ every 2 h. In a second set of NTHi growth experiments, three different concentrations of hemin (1 μm, 0.1 μm, and 0.01 μm) and *Ht*‐DM (1 μm, 0.1 μm, and 0.01 μm) were used to supplement BHI.

When using *Ht*‐C12A as a heme source, the proportion of heme covalently bound to the protein had to be considered. Prior work on *Ht*‐C12A revealed that ~14% of the sample had noncovalently bound heme, with the remainder covalently bound 17. To consider this factor, we supplemented BHI with a ‘high’ concentration of *Ht*‐C12A (6–7 μm) to test whether these conditions provided sufficient ‘free’ noncovalently bound heme for NTHi to grow 16. We also grew NTHi in BHI supplemented with a ‘low’ concentration of *Ht*‐C12A (0.6 μm). As positive controls, we grew NTHi in BHI supplemented with 6–7 μm hemin and 0.6 μm hemin. All heme sources were diluted in 1 mL of water and added to 5 mL of sterile BHI and 30 μm NAD^+^. Growths were incubated (37 °C, 180–200 rpm) for 24 h. Readings (OD_490_) were recorded after 12 h of growth and then every 2 h.

Nontypeable *H. influenzae* were also grown on BHI using 15 μm hemin, 15 μm MP‐11 (Sigma), or water (negative control) as its heme source. The NTHi were cultured as described above, and the OD_490_ was monitored every 2 h.

## Results and Discussion

Nontypeable *H. influenzae* have a strict requirement for heme as their source of iron, thus providing biological motivation for their complex and redundant heme acquisition and uptake systems 4, 10, 12, 13, 14, 22. Hemophores play an important role in those systems. Secreted into the extracellular space or tethered to the bacterial surface, hemophores serve as the initial heme ‘scavengers’ by stealing heme from hemoproteins directly or by lowering the hemoprotein's affinity for heme. Cyts *c* are ubiquitous hemoproteins, highly conserved across species, and found in most living organisms. Defined, in part, by their two covalent thioether linkages to heme, cyts *c* exhibit a diverse array of cellular functions in such biological arenas as energy transduction and apoptosis 23. Our *Ht* cyt *c* variants, with one, two, or zero covalent linkages to heme, provide a means to test what requirements and limitations there are on heme acquisition by NTHi with respect to the mode of heme binding to polypeptide.

**Figure 2:**
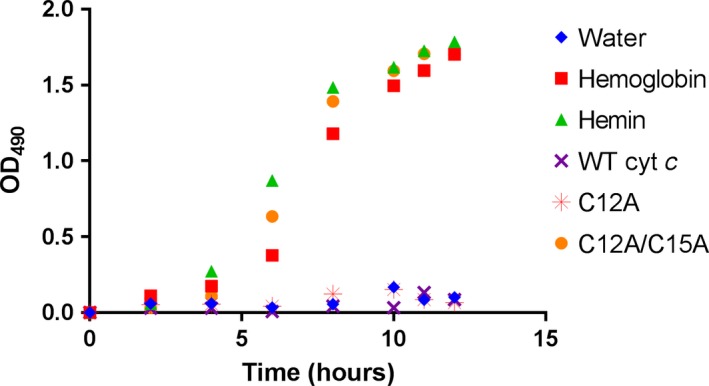
Nontypeable *Haemophilus influenzae* growth curves, monitored at 490 nm, show that NTHi cultures grow in BHI supplemented with 15 μm hemoglobin, hemin, or *Ht*‐DM (C12A/C15A), but do not grow with water, 15 μm wild‐type *Ht* cyt *c*, or 15 μm *Ht*‐C12A.

To determine the ability of NTHi to scavenge heme from *Ht* cyt *c* and variants with one or two thioether linkages removed, we cultured NTHi in BHI, supplemented with 30 μm NAD^+^ and (a) sterile water (negative control); (b) 15 μm hemoglobin; (c) 15 μm hemin (positive control); (d) 15 μm wild‐type *Ht* cyt *c*; (e) 15 μm *Ht*‐C12A; or (f) 15 μm *Ht*‐DM. For all experiments, hemin was used as a positive control. Hemoglobin contains noncovalently bound heme 24. Growth was tracked by measuring the OD at 490 nm every 2 h. NTHi showed minimal growth when BHI was supplemented with sterile water, wild‐type *Ht* cyt *c*, or *Ht*‐C12A (Fig. 2). In comparison, NTHi grew to log phase and approached stationary phase by hour twelve when BHI was supplemented with hemoglobin, hemin, or *Ht*‐DM (where both thioether linkages were absent) (Fig. 2). Two additional independent growth experiments using the same parameters and supplements yielded similar results (Fig. S1), which suggests that NTHi hemophore(s) can access and extract heme from *Ht*‐DM.

**Figure 3:**
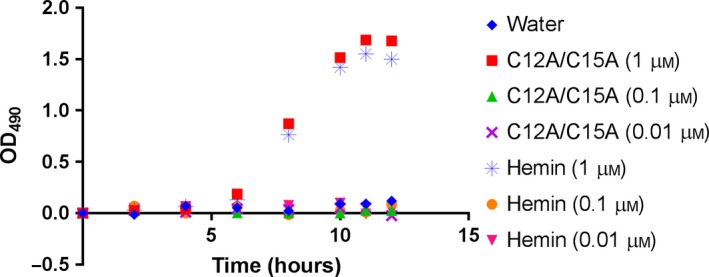
Growth curves, monitored at 490 nm, are similar for NTHi cultured in BHI supplemented with 1 μm hemin and 1 μm *Ht*‐DM (C12A/C15A); lower concentrations (0.1 μm or 0.01 μm) of hemin or *Ht*‐DM do not support NTHi growth.

In a second growth study, we determined that 1 μm hemin supports NTHi growth, while 0.1 μm hemin is insufficient for NTHi growth. We observed similar growth trends for the equivalent concentrations of *Ht*‐DM (Fig. 3), indicating that heme noncovalently bound to this protein scaffold supports growth as well as hemin. Two additional independent growth experiments using the same parameters and supplements yielded similar results (Fig. S2), which suggest that both of these heme sources (hemin and *Ht*‐DM) are similarly accessible to NTHi hemophore(s). In contrast to NTHi, which we found require 0.1–1.0 μm hemin for unrestricted growth, Hib requires a minimum of 0.03 μm free heme for unrestricted growth, indicating different heme requirements for these bacteria in culture 15. Further, we predict that NTHi cultured under different conditions will have different heme requirements, not surprisingly as *H. influenzae* has been shown to exhibit different heme requirements in the presence and absence of oxygen 25. As NTHi are pathogens (causing AOM in the inner ear and bronchitis and COPD in the lungs) and commensal organisms (in the nasal cavity), these nuances in NTHi's heme requirements may come into play as the bacteria live and thrive in different environments.

**Figure 4:**
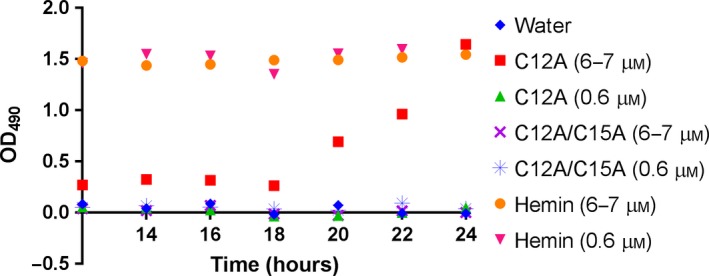
Nontypeable *Haemophilus influenzae* growth curves, monitored at 490 nm after 12 h of growth. NTHi cultures in BHI were supplemented with high (6–7 μm) or low (0.6 μm) concentrations of hemin, *Ht*‐C12A, or *Ht*‐C15A. NTHi grew with both concentrations of hemin and grew slowly with high concentrations of *Ht*‐C12A.

In *Ht*‐C15A, only the Cys12 residue is able to covalently link to the heme group, while in *Ht*‐C12A, only the Cys15 residue covalently links to the heme. However, it has been shown that the Cys15‐to‐heme covalent bond does not form in 100% of the *Ht*‐C12A protein sample, when overexpressed in *E. coli* 16. Specifically, Tomlinson and Ferguson showed that the heme group in *Ht*‐C12A is covalently bound in 86% of the sample, while the heme in *Ht*‐C15A is bound in 100% of the sample 16. To determine whether the small proportion of noncovalently bound heme in *Ht*‐C12A could support NTHi growth, we cultured NTHi in the presence of each single mutant at high concentrations. Results from our previous experiment suggested that NTHi requires between 0.1 μm and 1 μm accessible heme to grow. Assuming ~14% of *Ht*‐C12A contains noncovalently bound heme, we estimated that we would need to supplement BHI with between 0.7 μm and 7 μm of *Ht*‐C15A to observe growth similar to what was seen using excess hemin. As such, we grew NTHi in the presence of either high (6–7 μm) or low (0.6 μm) concentrations of hemin, *Ht*‐C12A, or *Ht*‐C15A 16. NTHi cultures reached log phase in less than 12 h when supplemented with high and low concentrations of hemin, but did not grow in the presence of the negative control, high or low concentrations of *Ht*‐C15A, or low concentrations of *Ht*‐C12A. When supplemented with high concentrations of *Ht*‐C12A, NTHi grew slowly, but eventually reached similar OD_490_ levels to the hemin‐supplemented growths by 24 h (Fig. 4). Two additional independent growth experiments using the same parameters and supplements yielded similar results (Fig. S3). The logarithmic growth phase was not reached until approximately 18 h. The delay in growth may have been the result of the noncovalently bound heme in *Ht*‐C12A being less accessible to NTHi than the equivalent concentration of hemin.

**Figure 5:**
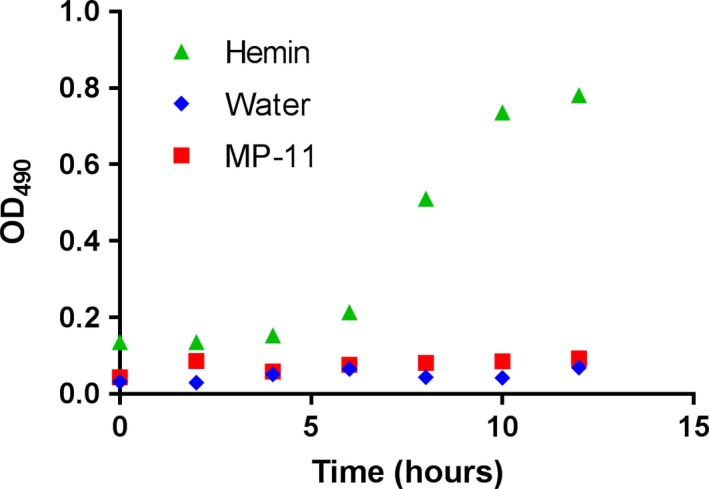
Nontypeable *Haemophilus influenzae* growth curves, monitored at 490 nm; NTHi cultures in BHI were supplemented with 15 μm hemin, water, or 15 μm MP‐11.

Microperoxidase‐11 (MP‐11) is a heme peptide derived from proteolysis of horse heart cyt *c* 18. The heme in MP‐11 is covalently attached to an 11‐mer peptide via the two Cys residues contained within the CXXCH motif, and the heme iron is coordinated to a single His residue donated by the same sequence. If heme uptake requires binding to a hemophore or receptor protein in a ‘tight’ pocket, as described for the heme transport protein HbpA, even a small attached peptide could prevent proper binding/fitting of the heme 26. Additionally, the coordinated His of MP‐11 may not be readily displaced, because it is positioned in the covalently bound peptide; this His–heme interaction could also hinder its interaction with a hemophore. In order to determine whether MP‐11 can serve as a heme source for NTHi, we repeated the initial NTHi growth experiment, using hemin (positive control), MP‐11, or water (negative control) as the heme source. Results from the experiment suggest that the covalent attachment of the heme to the 11‐mer peptide prevents MP‐11 from serving as a heme source for NTHi (Fig. 5). Two additional independent growth experiments using the same parameters and supplements yielded similar results (Fig. S4).

## Conclusions

Nontypeable *H. influenzae* are known to colonize in the nasopharynx of humans. Considering their requirement for heme, NTHi must acquire heme from human proteins such as hemoglobin or complexes such as heme bound to hemopexin. Cyts *c*, containing two covalent linkages to heme, have failed to serve as a heme source for *H. influenzae*. Here, we showed that even a single thioether bond between heme and polypeptide is sufficient to prevent heme scavenging from *Ht* cyt *c* mutants by NTHi. However, NTHi hemophores can interact with and extract heme from *Ht*‐DM, which contains zero covalent linkages to heme. We also confirmed that the C15‐to‐heme bond does not form 100% of the time in *Ht*‐C12A, allowing for heme scavenging by NTHi from high concentrations of this variant. Finally, we showed that MP‐11, which is similar in structure to hemin, but covalently tethered to an 11‐mer peptide, does not function as a heme source for NTHi. Results from this study suggest that the only accessible protein‐associated heme to support NTHi growth is noncovalently bound heme.

## Author contributions

VS, BN, SS, JP, BK, MFH, and KG collected experimental data and assisted with data analysis. VS, KLB, and LVM designed the study, analyzed data, and prepared the manuscript. KLB and LVM provided reagents.

## Supporting information


**Fig. S1.** Two additional NTHi growth curves, monitored at 490 nm, show that NTHi grows in BHI supplemented with hemoglobin, hemin, or *Ht*‐DM (C12A/C15A), but does not grow with water, wild‐type *Ht* cyt *c*, or single mutant *Ht*‐C12A.
**Fig. S2.** Two additional NTHi growth curves, monitored at 490 nm, show that NTHi grows in BHI supplemented with 1 μm hemin or *Ht*‐DM (C12A/C15A), but does not grow with lower concentrations of hemin or *Ht*‐DM.
**Fig. S3.** Two additional NTHi growth curves, monitored at 490 nm after 12 h of growth.
**Fig. S4.** Two additional NTHi growth curves, monitored at 490 nm, show that NTHi grows in BHI supplemented with 15 μm hemin, but does not grow with water or 15 μm MP‐11.
