# Supplementary material for: Covalent bonding of heme to protein prevents heme capture by nontypeable Haemophilus influenzae
Source: FEBS Open Bio. 2017 Oct 12;7(11):1778–83. doi: 10.1002/2211-5463.12324 (PMC5666386; doi:10.1002/2211-5463.12324)
Supplement: Supplementary file 1 — Fig. S1. Two additional NTHi growth curves, monitored at 490 nm, show that NTHi grows in BHI supplemented with hemoglobin, hemin, or Ht‐DM (C12A/C15A), but does not grow with water, wild‐type Ht cyt c, or single mutant Ht‐C12A. Fig. S2. Two additional NTHi growth curves, monitored at 490 nm, show that NTHi grows in BHI supplemented with 1 μm hemin or Ht‐DM (C12A/C15A), but does not grow with lower concentrations of hemin or Ht‐DM. Fig. S3. Two additional NTHi growth curves, monitored at 490 nm after 12 h of growth. Fig. S4. Two additional NTHi growth curves, monitored at 490 nm, show that NTHi grows in BHI supplemented with 15 μm hemin, but does not grow with water or 15 μm MP‐11. [file FEB4-7-1778-s001.pdf]

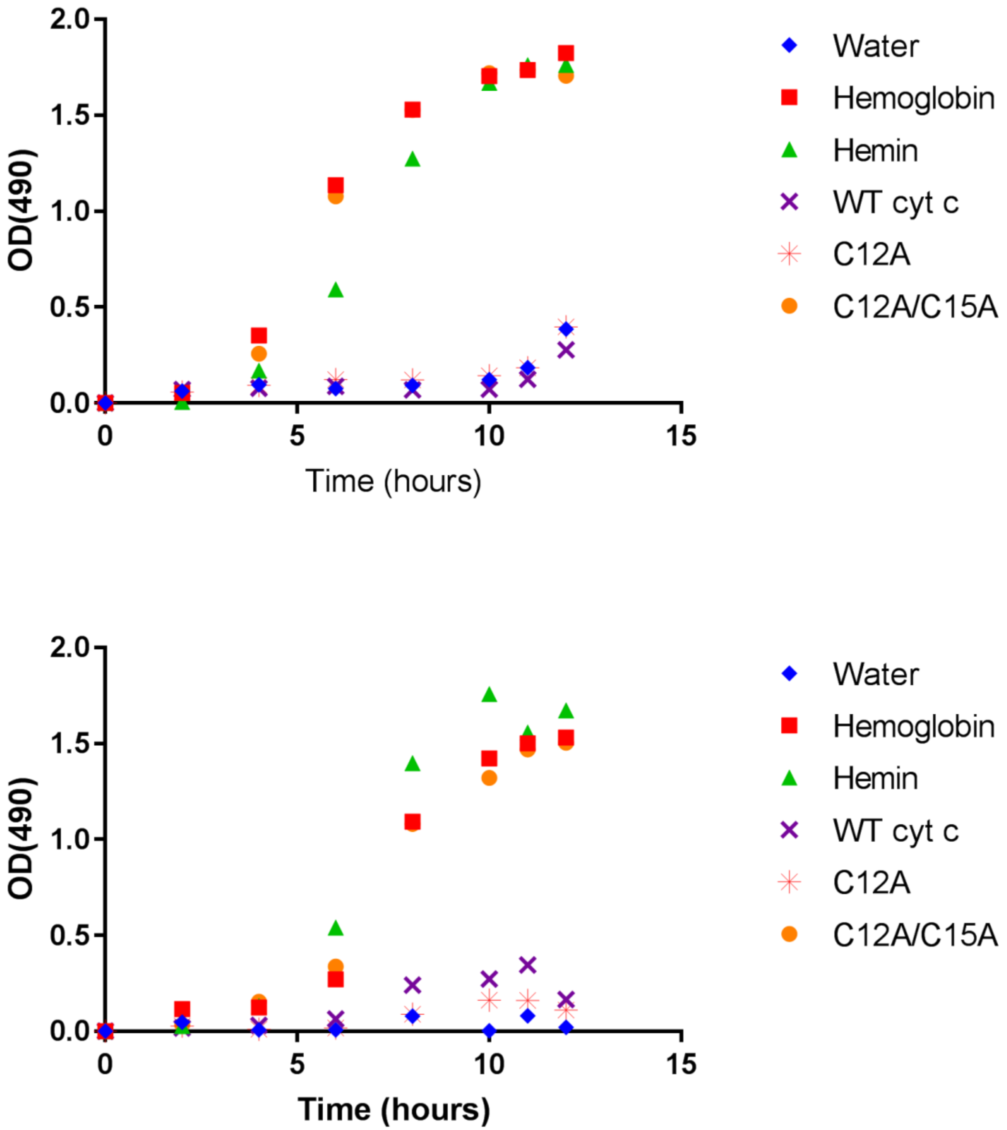

Supplementary Figure 1. Two additional NTHi growth curves, monitored at 490 nm, show that NTHi grows in BHI supplemented with hemoglobin, hemin, or *Ht*-DM (C12A/C15A), but does not grow with water, wild-type *Ht* cyt *c*, or single mutant *Ht*-C12A.

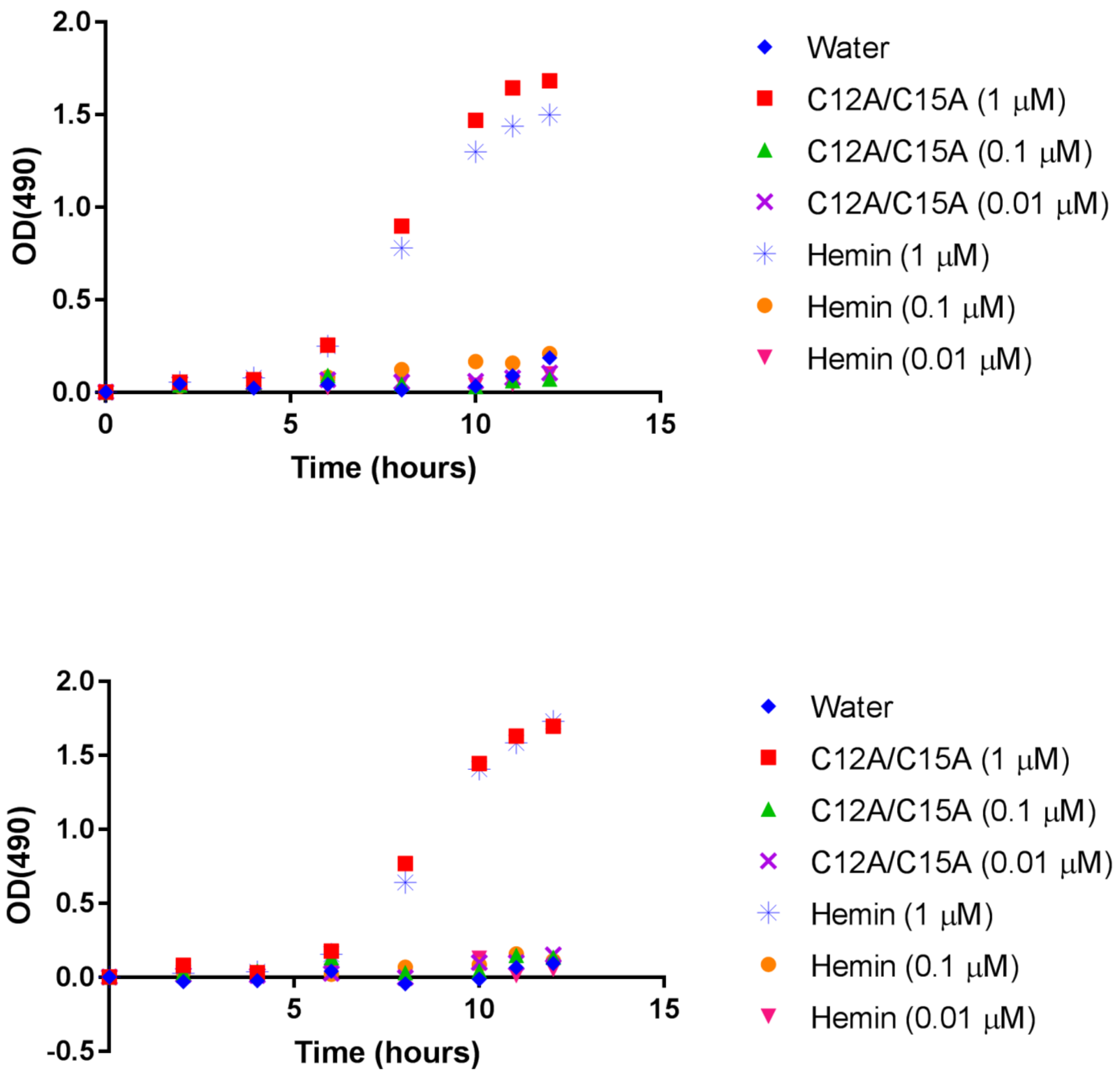

Supplementary Figure 2. Two additional NTHi growth curves, monitored at 490 nm, show that NTHi grows in BHI supplemented with 1 μM hemin or *Ht*-DM (C12A/C15A), but does not grow with lower concentrations of hemin or *Ht*-DM.

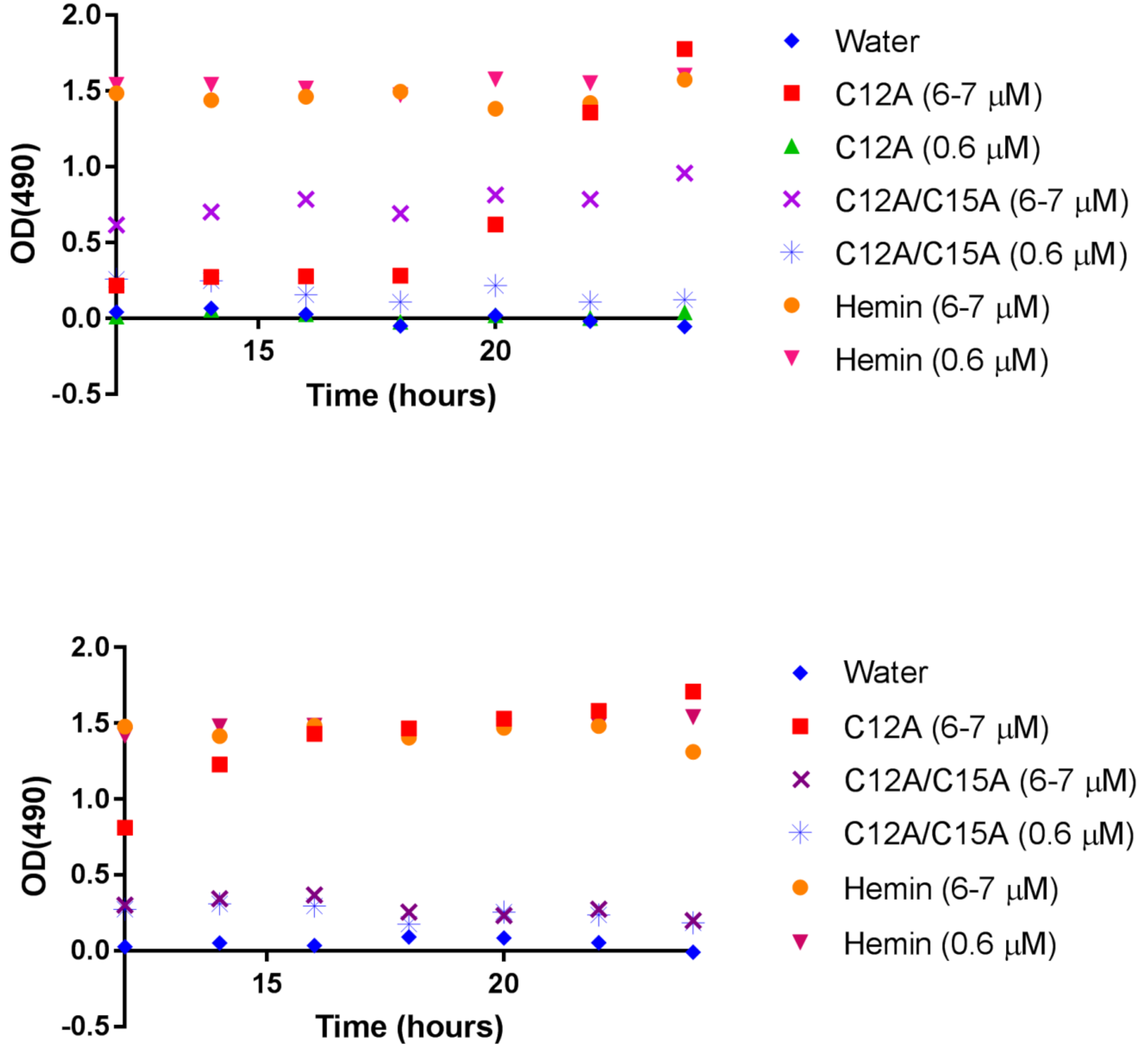

Supplementary Figure 3. Two additional NTHi growth curves, monitored at 490 nm after 12 hours of growth. BHI media was supplemented with NAD^+ and either high (6-7 mM) or low (0.6 mM) concentrations of hemin, *Ht*-C12A, or *Ht*-C15A. NTHi grows with both concentrations of hemin and grows slowly when supplemented with high concentrations of *Ht*-C12A. In the second experiment (bottom plot), the low concentration C12A sample was visibly contaminated at 12 hours, so was discarded and excluded from the final plot.

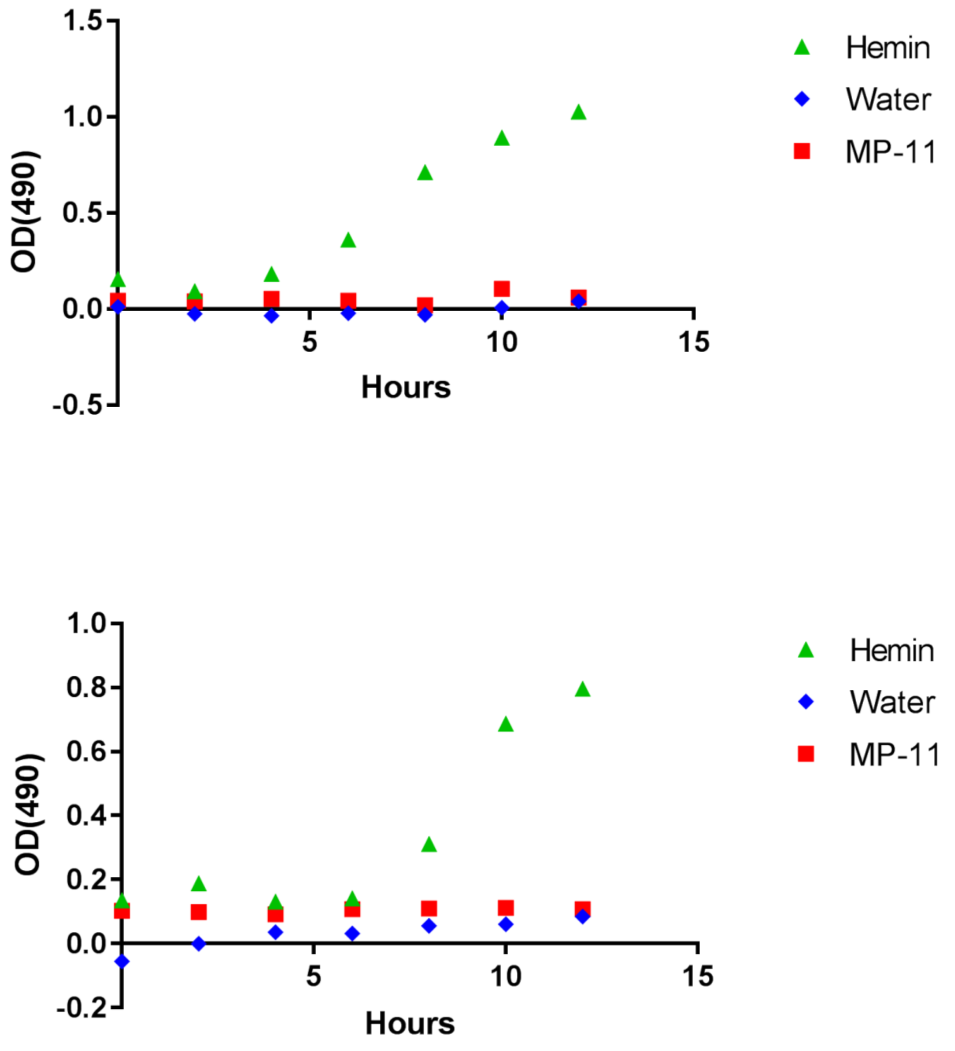

Supplementary Figure 4. Two additional NTHi growth curves, monitored at 490 nm, show that NTHi grows in BHI supplemented with 15 μM hemin, but does not grow with water or 15 μM MP-11.
